# Maternal Blood Fatty Acid Levels in Fetal Growth Restriction

**DOI:** 10.1055/s-0043-1768455

**Published:** 2023-04-27

**Authors:** Raquel Margiotte Grohmann, Vivian Macedo Gomes Marçal, Isabela César Corazza, Alberto Borges Peixoto, Edward Araujo Júnior, Luciano Marcondes Machado Nardozza

**Affiliations:** 1 Department of Obstetrics, Paulista School of Medicine–Federal University of São Paulo, São Paulo, SP, Brazil; 2 Department of Obstetrics and Gynecology, Medical Science College of Santos, Santos, SP, Brazil; 3 Department of Gynecology and Obstetrics, Federal University of Triângulo Mineiro, Uberaba, MG, Brazil; 4 Gynecology and Obstetrics Service, Mário Palmério University Hospital, University of Uberaba, Uberaba, MG, Brazil

**Keywords:** Fetal growth restriction, Maternal blood, Appropriate for gestational age, Fatty acids, Restrição de crescimento fetal, Sangue materno, Adequado para idade gestacional, Ácidos graxos

## Abstract

**Objective:** To assess the maternal blood levels of fatty acids (FAs) in pregnancies with fetal growth restriction (FGR).

**Methods:** This prospective cross-sectional study included pregnant women with gestational age between 26 and 37 + 6 weeks with FGR and appropriate for gestational age (AGA) fetuses. The levels of saturated, trans, monounsaturated, and polyunsaturated FAs were measured using centrifugation and liquid chromatography. The Student's t *-* test, Mann–Whitney test, and general linear model, with gestational age and maternal weight as covariants, were used to compare FA levels and the FGR and AGA groups. The Chi-square was used to evaluate the association between groups and studied variables.

**Results:** Maternal blood sample was collected from 64 pregnant women, being 24 FGR and 40 AGA. A weak positive correlation was found between the palmitoleic acid level and maternal weight (r = 0.285, *p* = 0.036). A weak negative correlation was found between the gamma-linoleic acid level and gestational age (r = − 0.277, *p* = 0.026). The median of the elaidic acid level (2.3 vs. 4.7 ng/ml, *p* = 0.045) and gamma-linoleic acid (6.3 vs. 6.6 ng/ml, *p* = 0.024) was significantly lower in the FGR than the AGA group. The palmitoleic acid level was significantly higher in the FGR than AGA group (50.5 vs. 47.6 ng/ml, *p* = 0.033).

**Conclusion:** Pregnant women with FGR had lower elaidic acid and gamma-linoleic acid levels and higher palmitoleic acid levels than AGA fetuses.

## Resumo

**Objetivo:** Avaliar os níveis sanguíneos maternos de ácidos graxos (AGs) em gestações com restrição de crescimento fetal (RCF).

**Métodos:** Este estudo prospectivo transversal incluiu gestantes com idade gestacional entre 26 e 37 semanas e 6 dias com RCF e fetos adequados para a idade gestacional (AIG). Os níveis de ácidos graxos saturados, trans, monoinsaturados e poliinsaturados foram medidos usando centrifugação e cromatografia líquida. O teste t-Student, o teste de Mann-Whitney e o modelo linear geral, com idade gestacional e peso materno como covariantes, foram utilizados para comparar os níveis de AGs e os grupos RCF e AIG. O teste Qui-quadrado foi utilizado para avaliar a associação entre os grupos e as variáveis estudadas.

**Resultados:** Amostra de sangue materno foi coletada de 64 gestantes, sendo 24 RCF e 40 AIG. Uma correlação positiva fraca foi encontrada entre o nível de ácido palmitoleico e o peso materno (r = 0,285, p = 0,036). Uma correlação negativa fraca foi encontrada entre o nível de ácido gama-linoleico e a idade gestacional (r = − 0,277, p = 0,026). A mediana do nível de ácido elaídico (2,3 vs. 4,7 ng/ml, p = 0,045) e ácido gama-linoleico (6,3 vs. 6,6 ng/ml, p = 0,024) foram significativamente menores no grupo RCF do que no grupo AIG. O nível de ácido palmitoleico foi significativamente maior no grupo RCF do que no grupo AIG (50,5 vs. 47,6 ng/ml, p = 0,033).

**Conclusão:** Gestantes com RCF apresentaram níveis mais baixos de ácido elaídico e ácido gama-linoleico e níveis mais elevados de ácido palmitoleico do que os fetos AIG.

## Introduction

Fetal growth restriction (FGR) is an intercurrence that affects 5%–10% of pregnancies, the second leading cause of perinatal mortality, and is responsible for approximately 30% of stillbirths, in addition to determining a higher frequency of premature births and intrapartum asphyxia. 1

Nowadays, there is no effective treatment to decrease or stop placental insufficiency progression, thus fetal vitality assessment and the decision regarding the delivery are the main strategies in the management of these fetuses. 2 Within this context, finding an effective, non-invasive, and low-cost treatment would be important to decrease the FGR rates. Maternal dietary supplementation with omega-3 polyunsaturated fatty acids (PUFAs) during pregnancy has been shown to increase gestational duration, increase fetal growth, and decrease the risk of pregnancy complications, although its precise mechanisms remain uncertain. 3

Fatty acids (FAs) are long-chain organic acids and basic compounds of lipids, which are classified into saturated and unsaturated. Saturated FAs (SFAs) are those with single carbons bonds, mainly found in fat animal products in solid-state. Unsaturated FAs have carbons that make one or more double bonds exist mainly in vegetables in liquid form. Unsaturated FAs can be further classified into monounsaturated–with only one carbon double bond and polyunsaturated–with two or more double bonds. 4 5

Some benefits of maternal supplementation of omega-3 PUFAs have been described in the literature, such as reduction of depression during pregnancy and after delivery 6 and decreased preterm birth in pregnant women with low total omega-3 PUFA status early in pregnancy. 6 7 However, whether omega-3 PUFAs interfere with fetal growth remains unclear, particularly in those cases with FGR. Therefore, establishing the relationship between a diet rich in omega-3 PUFAs and fetal growth is necessary to elucidate the anti-inflammatory power of this FA in this disease.

This study aimed to compare the maternal blood levels of FAs in pregnancies with FGR and appropriate for gestational age (AGA) fetuses.

## Methods

This cross-sectional study was conducted between February 2017 and May 2021 and approved by the Research Ethics Committee of the Federal University of São Paulo (UNIFESP) (n: 2.004.104). All participants signed the consent form. During this period, FAs were analyzed in pregnant women who were divided into two groups: 1) early- and late-onset FGR and 2) AGA. Fetal growth restriction (FGR) was defined according to the Delphi criteria. 8 Early-onset FGR - gestational age (GA) was <32 weeks and estimated fetal weight (EFW) or abdominal circumference (AC) < 3rd percentile for the GA 9 or absent end-diastolic flow in the umbilical artery (UA) Doppler, EFW or AC < 10 ^th^ percentile for the GA 9 associated with a mean pulsatility index (PI) of uterine artery Doppler or PI UA Doppler > 95th percentile for the GA. 10 11 Late-onset FGR - GA >32 weeks and EFW or AC < 3rd percentile for the GA, 9 EFW or AC < 10 ^th^ percentile for the GA 9 associated with a mean PI UA Doppler > 95th percentile for the GA, 11 cerebral placental ratios < 5th percentile for the GA, 12 or AC and/or EFW crossing centiles of >2 quartiles on growth centiles. Appropriate for gestational age (AGA) was if the values come from

10th and 90th percentiles according to the table proposed by Hadlock et al. 9

Following the FGR diagnosis, pregnant women were assessed at the Fetal Growth Restriction Sector of the Department of Obstetrics, UNIFESP. The inclusion criterion was singleton pregnancy with GA between 26 and 37 + 6 weeks confirmed by ultrasonography performed up to the 13 ^th^ week. The exclusion criteria were pregnant women in labor and fetuses with structural malformations and/or chromosomal disorders.

The following FAs were assessed: 1) SFAs: myristic, palmitic, and stearic acids; 2) trans FA (TFA): elaidic acid; 3) monounsaturated FAs: palmitoleic and oleic acids; 4) omega-6 PUFAs: linoleic, dihomo gamma-linoleic, arachidonic, and gamma-linoleic acids; and 5) omega-3 PUFAs: alpha-linoleic, eicosapentaenoic, and docosahexaenoic acids.

Maternal blood samples were collected, centrifuged, and sent for laboratory analysis within 24 h. Fatty acid levels were assessed according to the methodology proposed by Kolarovic and Fournier. 13 This method consisted of initially extracting the total lipid from the plasma. Briefly, 500 ul of plasma was mixed with 500 ul of water by Vortex for 30 sec with 100 ul “internal standard” containing 0.857 mg of heptadecanoic acid/ml as a phospholipid dissolved in chloroform. A mixture of hexane and 2-propanol at 4 ml containing 25 mg of di-tert-butyl methyl phenol was added.

Phospholipids were isolated by liquid chromatography using an aminopropyl column (Sep Pak Cartridges; Waters, Milford, MA) as described by Agren et al. 14 The phospholipid fractions obtained on the columns were vacuum dried, and each well was added with 100 µl of chloroform. Fatty acid methyl ester was formed according to the method by Lepage et al. 15 A gas chromatograph (model HP-5890 Series II; Hewlett-Packard, Palo Alto, CA) equipped with a flame ionization detector was used to quantify FA methyl esters.

Chromatography was performed using a 60-m wide capillary column, 0.32-mm internal diameter, and 20-um film thickness (Sp 2330FS; Supelco Inc, Bellefonte, Palo Alto, CA). A 29:1 split ratio injector and detector were maintained at 250°C and 275°C, respectively, and nitrogen was used as a carrier gas. The docosahexaenoic and eicosapentaenoic acid proportions were calculated as a weight percentage (% by weight) of the total detected FAs with 14–24 carbon atoms.

Maternal eating habits and smoking variables were collected using a standardized questionnaire which was applied before the collection of blood samples.

To evaluate the effect of FGR and AGA fetuses on the levels of maternal FAs, a power analysis was performed to calculate the sample size on the basis of the Cohen effect of 0.7 to achieve a power of 80% and an alpha of 5% to detect the differences in the evaluated parameters. 16 Using the software G 3.1, the results suggested a total sample size of 62 pregnant women.

Data were collected in an Excel 2007 spreadsheet (Microsoft Corp., Redmond, WA, USA) and analyzed using statistical software Statistical Package for the Social Sciences version 15.0 (SPSS Inc., Chicago, IL, USA) and Prisma GraphPad version 7.0 (GraphPad Software; San Diego, CA, USA). The D'Agostino and Pearson normality test was used to analyze if the values come from the Gaussian distribution. The non-parametric distribution variables were presented as medians and interquartile ranges. The normal distribution variables were presented as mean and standard deviation. Categorical variables were described as absolute and percentage frequencies and represented in Tables. The Student's t-test, Mann-Whitney test, and general linear model, with GA and maternal weight as covariant, were used to compare the FA levels between the groups. The correlation between the FA levels, GA, and maternal weight was performed using the Pearson and Spearman tests. The Chi-square test was used to study the difference between categorical variables and their proportions. General linear regression was performed to assess the ability of gestational age to predict FA levels in the maternal blood sample. A *p* -value of <0.05 was considered statistically significant.

## Results

Maternal blood samples were collected from 67 pregnant women; however, 3 were excluded from the analysis due to blood coagulation (n = 2) and gestational age beyond the period (n = 1). The maternal characteristics, such as maternal age, parity, height, weight, body mass index (BMI), gestational age, and EFW on the day of maternal blood sample collection, are shown in Table 1 . The mean EFW was significantly lower in the FGR than AGA fetuses (1,439.0 vs. 1,717.0 g, *p* = 0.041).

**Table 1:** Comparison of maternal characteristics between appropriate for gestational age (AGA) and fetal growth restriction (FGR) fetuses

Maternal Characteristics	FGR (N = 24)	AGA (N = 40)	*p* -value
Age (years)	23.0 (19.0–29.0)	22.5 (17.0–32.2)	0.561 ^†^
Parity			0.220 ^§^
Primiparous	52.6% (10/19)	72.4% (21/29)	
Multiparous	47.4% (9/19)	27.6% (8/29)	
Height (m)	1.59 (0.06)	1.62 (0.08)	0.132 ^∫^
Weight (kg)	65.2 (13.4)	69.4 (14.8)	0.292 ^∫^
BMI (kg/m ^2^ )	26.0 (4.8)	26.6 (4.4)	0.654 ^∫^
Gestational age (weeks)	32.2 (3.2)	31.3 (3.0)	0.259 ^∫^
Estimated fetal weight (grams)	1439.0 (461.3)	1717.0 (537.9)	0.041 ^∫^

BMI: body mass index. Mann-Whitney †: median (interquartile range); Student's t-test ∫: mean (standard deviation); Chi-square §: Percentage (n/N). *p* < 0.05, statistically significant.

**Fig. 1:**
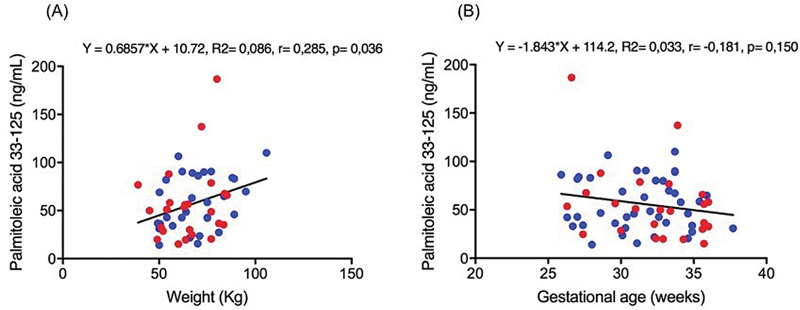
Correlation between the maternal palmitoleic acid levels with appropriate for gestational age (blue dots), fetal growth restriction (red dots), maternal weight (A), and gestational age (B). Spearman test, *p* < 0.05.

Considering all cases, a weak positive correlation was found between the palmitoleic acid level and maternal weight (r = 0.285, *p* = 0.036). No significant correlation was found between the palmitoleic acid level and gestational age (r = − 0.181, *p* = 0.150) ( Fig. 1 ). Although significant, only 8.6% of the palmitoleic acid level was linearly related to maternal weight. The increased maternal weight of 1.0 kg was responsible for increasing the palmitoleic acid level by 0.68 ng/ml. No significant correlation was found between the other studied saturated, trans, and monounsaturated FA levels and maternal weight ( Supplementary Material Figure S1 ). No significant correlation was found between the studied saturated, trans, and monounsaturated FA levels and gestational age ( Supplementary Material Figure S2 ).

Table 2 shows the comparison of the saturated, trans, and monounsaturated FA levels in FGR and AGA fetuses. The median elaidic acid level was significantly lower in FGR than in AGA fetuses (2.3 vs. 4.7 ng/ml, *p* = 0.045). The median palmitoleic acid level was significantly higher in FGR than in AGA fetuses using the maternal weight as a covariant (50.5 vs. 47.6 ng/ml, *p* = 0.033).

**Table 2:** Maternal fatty acids levels with fetal growth restriction (FGR) and appropriate for gestational age (AGA) fetuses

Fatty acids	FGR (n = 24)	AGA (n = 40)	*p-value*
Myristic acid 15–60 (ng/mL)	22.6 (18.5–42.9)	28,8 (20–41.8)	0.375 ^†^
Palmitic acid 320–1075 (ng/mL)	704.6 (277.3)	738.0 (253.5)	0.624 ^∫^
Stearic acid 127–305 (ng/mL)	165.5 (54.3)	174.4 (51.1)	0.514 ^∫^
Elaidic acid <10 (ng/mL)	2.3 (1.8–5.4)	4.7 (2.85–6.8)	0.045 ^†^
Palmitoleic acid 33–125 (ng/mL)	50.5 (29.0–67.1)	47.6 (33.9–81.5)	0.033 ^§^
Oleic acid 260–1250 (ng/mL)	469.2 (320.2–624.5)	522.1 (403.8–631.1)	0.315 ^†^

Mann-Whitney †: median (interquartile range); Student's t-test ∫: mean (standard deviation); General Linear Model with maternal weight as covariant §: median (interquartile range). *p* < 0.05, statistically significant.

**Fig. 2:**
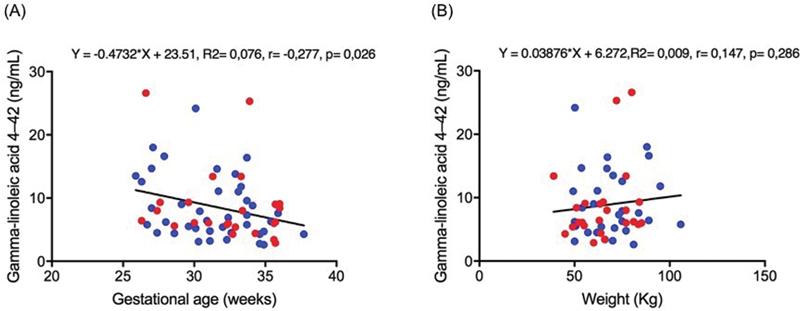
Correlation between the maternal gamma-linoleic acid levels with appropriate for gestational age (blue dots), fetal growth restriction (red dots), gestational age (A), and maternal weight (B). Spearman test, *p* < 0.05.

Considering all cases, a weak negative correlation was found between the gamma-linoleic acid level and gestational age (r = − 0.277, *p* = 0.026). No significant correlation was found between the gamma-linoleic acid level and maternal weight (r = 0.147, *p* = 0.286) ( Fig. 2 ). Although significant, only 7.6% of the gamma-linoleic acid level was linearly related to the gestational age. The increased gestational age of 1 week was responsible for decreasing the gamma-linoleic acid level by 0.47 ng/ml. No significant correlation was found between other omega-6 polyunsaturated FA levels and gestational age ( Supplementary Material Figure S3 ). No correlation was found between the omega-6 PUFA levels and maternal weight ( Supplementary Material Figure S4 ).

Table 3 shows the comparison of the omega-6 PUFA levels in FGR and AGA fetuses. The median gamma-linoleic acid level was significantly lower in FGR than in AGA fetuses using the maternal weight as a covariant (6.3 vs. 6.6 ng/ml, *p* = 0.024) ( Table 3 ).

**Table 3:** Maternal omega-6 polyunsaturated fatty acid levels with appropriate for gestational age (AGA) and fetal growth restriction (FGR) fetuses

Omega-6 polyunsaturated fatty acid	FGR (n = 24)	AGA (n = 40)	*p* -value
Linoleic acid 576–1300 (ng/mL)	895.5 (737.9–1110)	928.5 (773.7–1237)	0.284 ^†^
Dihomo gamma-linoleic acid 38–105 (ng/mL)	57.9 (40.1–81.6)	61.8 (47.7–78.4)	0.579 ^†^
Arachidonic acid 108–422 (ng/mL)	239.6 (196.8–291.4)	240.0 (181.3–309.7)	0.769 ^†^
Gamma-linoleic acid 4–42 (ng/mL)	6.3 (5.6–9.2)	6.6 (4.5–11.6)	0.024 ^§^

Mann-Whitney †: median (interquartile range). General linear model with gestational age as covariant §: median (interquartile range). *p* < 0.05, statistically significant.

Considering all cases, no significant correlation was found between the maternal omega-3 PUFA levels and maternal weight ( Supplementary Material Figure S5 ), as well as the gestational age ( Supplementary Material Figure S6 ). Table 4 shows the comparison of maternal omega-3 PUFA levels in FGR and AGA fetuses. No significant differences were found between the two groups regarding the omega-3 PUFA levels. Table 5 shows the comparison of maternal eating habits and smoking between FGA and AGA fetuses, which revealed no statistical differences.

**Table 4:** Maternal omega-3 polyunsaturated fatty acid levels with appropriate for gestational age (AGA) and fetal growth restriction (FGR) fetuses

Omega-3 polyunsaturated fatty acid	FGR (n = 24)	AGA (n = 40)	* p-value ^†^*
Alpha-linoleic acid 3–21 (ng/mL)	21.0 (14.5–31.7)	22.1 (18.0–29.1)	0.569
Eicosapentaenoic acid 5–73 (ng/mL)	5.5 (4.1–8.7)	8.3 (4.7–12.0)	0.111
Docosahexaenoic acid 34–160 (ng/mL)	87.0 (53.2–115.6)	85.4 (67.2–109.3)	0.841

Mann-Whitney †: median (interquartile range); *p* < 0.05, statistically significant.

**Table 5:** Maternal eating habits and smoking of appropriate for gestational age (AGA) and fetal growth restriction (FGR) fetuses

	AGA (n = 40)		FGR (n = 24)		p-value ^*^
Fish					0.751
No	33	82.5%	19	79.2%	
Yes	7	17.5%	5	20.8%	
Milk					0.543
No	8	20.0%	7	29.2%	
Yes	32	80.0%	17	70.8%	
Fry					0.589
No	12	30.0%	9	37.5%	
Yes	28	70.0%	15	62.5%	
Smoking					0.297
No	32	80.0%	22	91.7%	
Yes	8	20.0%	2	8.3%	
Olive oil					0.207
No	15	37.5%	13	54.2%	
Yes	25	62.5%	11	45.8%	

Chi-square *. *p* < 0.05, statistically significant.

## Discussion

Omega-3 PUFAs are beneficial in the regulation of maternal and fetal metabolic function, inflammation, immunity, macrosomia, oxidative stress, pre-eclampsia, FGR, preterm birth, offspring metabolic function, and neurodevelopment. 17 PUFAs have antioxidant activity, thus dietary supplementation of these substances during pregnancy has the potential to prevent or control placental disorders and promote fetal growth. 18 In our study, maternal FAs levels showed different behaviors in pregnant women with FGR and AGA fetuses.

Bobiński et al. 19 evaluated the maternal diet with AGA, preterm delivery, and small for gestational age (SGA) fetuses. Diet components were assessed by dietary questionnaire, and the authors concluded that the predictive factor was higher content of short- and medium-chain FAs in the maternal diet for AGA fetuses. In another study, Bobiński et al. 20 assessed the FA levels of the fetus (cordocentesis) and mothers who delivered full-term, SGA, and preterm newborns and concluded that the placental-fetal transport of FAs in full-term was different from SGA and preterm newborns. A previous study from our group compared the maternal blood levels of 40 SGA and 24 AGA fetuses and revealed no significant difference in SFAs, TFAs, monounsaturated, and PUFAs. 21 The present study is a secondary analysis of a larger study in which we compared SGA and FGR with the intention of evaluating whether placental insufficiency could interfere with maternal levels of FAs, given that SGA are investigated whether essential FA. As the sample sizes of both studies are similar, this could constitute a limitation of the study.

Our study revealed that maternal blood levels of TFAs (elaidic acid) and omega-6 PUFAs (gamma-linoleic acid) were lower in FGA than in AGA fetuses. Das 22 in a review article investigated whether essential FA metabolism and their long-chain metabolite concentrations (long-chain polyunsaturated FAs [LCPUFAs]) are altered in FGR. He revealed that low-birth-weight infants have decreased LCPUFA concentrations, especially arachidonic acid.

FAs modulate angiogenesis as observed by increased tube formation and angiogenic growth factor secretion in first-trimester human placental trophoblasts. During the third trimester of pregnancy, placental preferential transport of maternal plasma LCPUFAs is of critical importance for fetal growth and development. 23 Cetin et al. 24 assessed the fetal and maternal FA profiles in utero in 11 AGA and 10 FGR fetuses by cordocentesis between 19 and 39 weeks. Total plasma FA levels were significantly higher in the mother than in both AGA and FGR fetuses. The authors conclude that FGR could be related to inadequate transplacental supply as well as inadequate fetal enzymes for elaborating these metabolically relevant conditionally essential FAs.

Alvino et al. 25 compared the maternal FA levels between two groups (AGA, n = 42) and (FGR, n = 25). FGR was defined as AC measurement < 10 ^th^ percentile for GA. These authors observed that maternal total FA levels were similar between AGA and FGR, except the arachidonic acid/linoleic acid ratio which was These authors observed that maternal total FA significantly lower in FGR than in AGA fetuses.

Our study revealed no differences regarding eating habits and smoking between pregnant women with FGR and AGA fetuses. Middleton et al. 26 performed a systematic review including 70 randomized controlled trials and compared omega-3 LCPUFA interventions (supplements and food) with placebo or no omega-3 PUFAs. They revealed a reduced risk for low-birth-weight newborns; however, little or no difference in SGA and FGR. Saccone et al., 27 in a systematic review, assessed the maternal supplementation of LCPUFAs regarding perinatal outcomes, included 34 randomized controlled trials, and revealed that LCPUFA supplementation was not associated with obstetrical disorder prevention, such as preterm birth, pre-eclampsia, gestational diabetes mellitus, SGA, and FGR. Chen et al. 28 included 21 randomized controlled trials and revealed that fish oil supplementation was associated with higher birth weight, birth length, and head circumference, and a 23% lower risk of low-birth-weight. No benefit was found from fish oil supplementation about the risk of FGR or stillbirth.

For the correct analysis of all FAs assessed in the present study and their real interference in fetal growth and development, a rigorous analysis of the diet of pregnant women should have been established. Not only if the type of food ingested daily contained fat of animal or vegetable origin, as well as the intake of fish, vegetables, cocoa, olive oil, milk oils, nuts, coconut, among others, so common in our diet. The amount of these foods as well as their origin are essential to establish the correct influence of the diet in the determinism of FGR. A limitation in our study was not to accurately establish the diet of the participating pregnant women. As strength, it is the first study that compared the maternal FAs between AGA and FGR using a specific methodology.

## Conclusion

In summary, pregnant women with FGR had lower blood elaidic acid and gamma-linoleic acid levels and higher palmitoleic acid levels than AGA fetuses. Maternal eating habits and smoking did not show significant differences between FGR and AGA fetuses.
