# Supplementary material for: Maternal Blood Fatty Acid Levels in Fetal Growth Restriction
Source: Rev Bras Ginecol Obstet. 2023 Apr 27;45(3):127–33. doi: 10.1055/s-0043-1768455 (PMC10139773; doi:10.1055/s-0043-1768455)
Supplement: Supplementary file 1 — Supplementary Material [file 10-1055-s-0043-1768455-s220121.pdf]

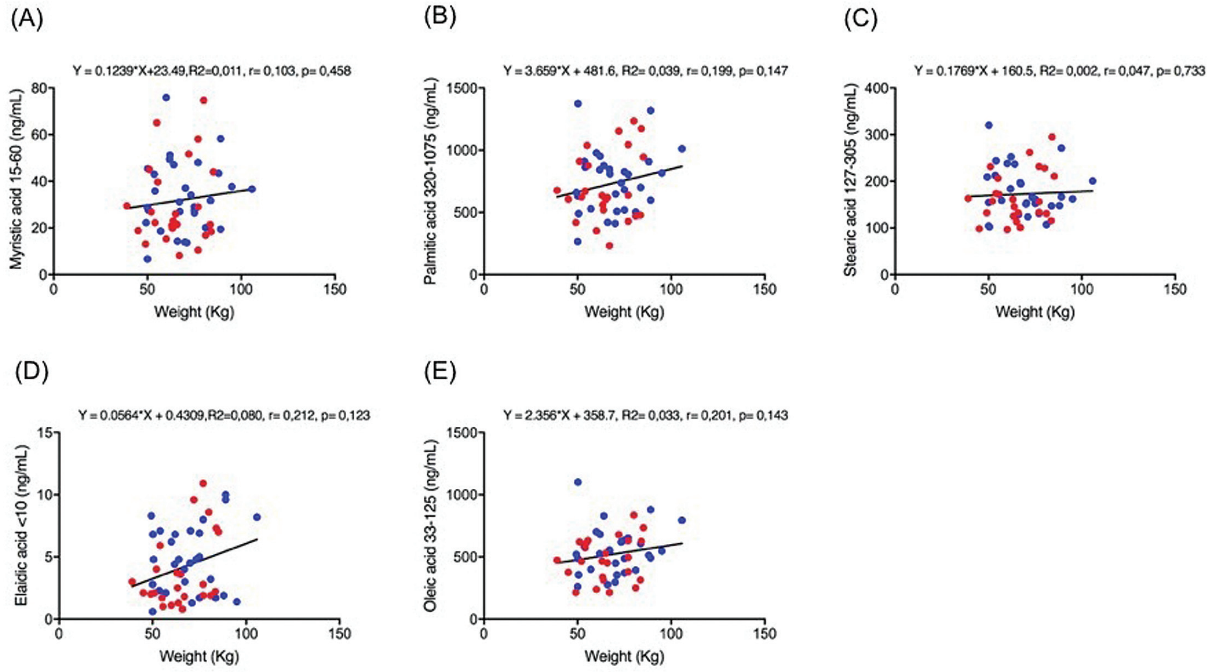

Supplementary Material Figure S1. Correlation between the saturated, trans, and monounsaturated fatty acid levels measured in pregnant women with appropriate for gestational age (blue dots), fetal growth restriction (red dots), and maternal weight. Pearson and Spearman test, $p < 0.05$

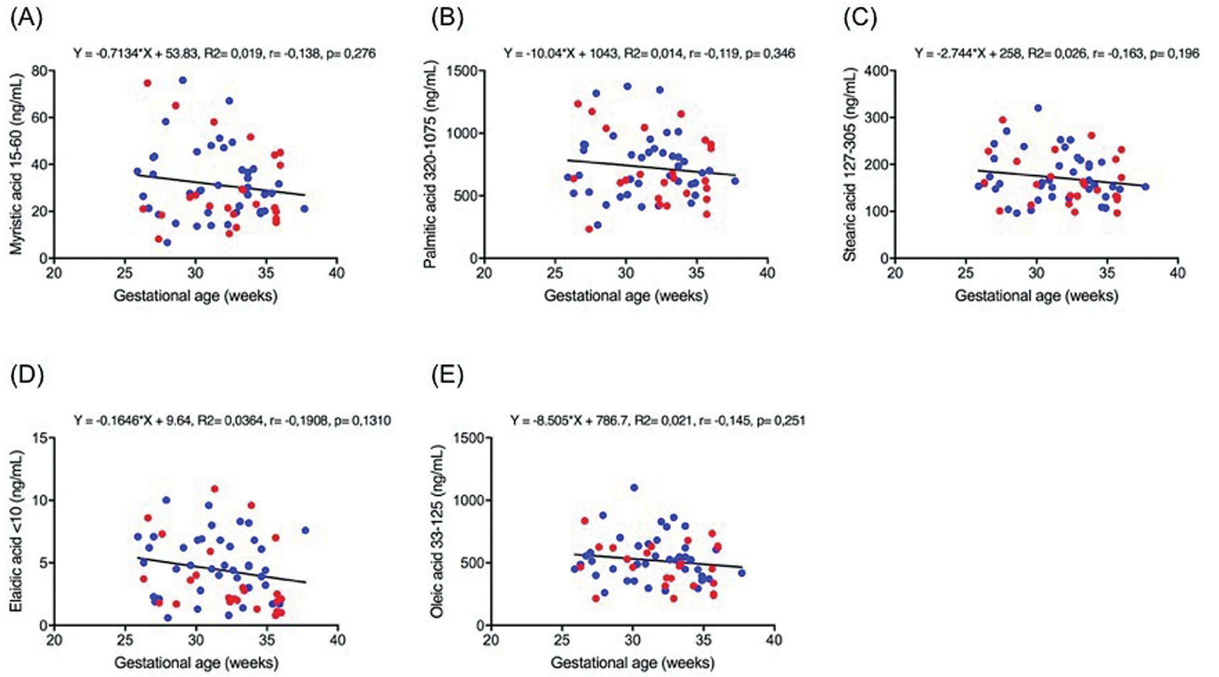

Supplementary Material Figure S2. Correlation between the saturated, trans, and monounsaturated fatty acid levels measured in pregnant women with appropriate for gestational age (blue dots), fetal growth restriction (red dots), and gestational age. Pearson and Spearman test, $p < 0.05$

 © 2023. Federação Brasileira de Ginecologia e Obstetrícia. All rights reserved.

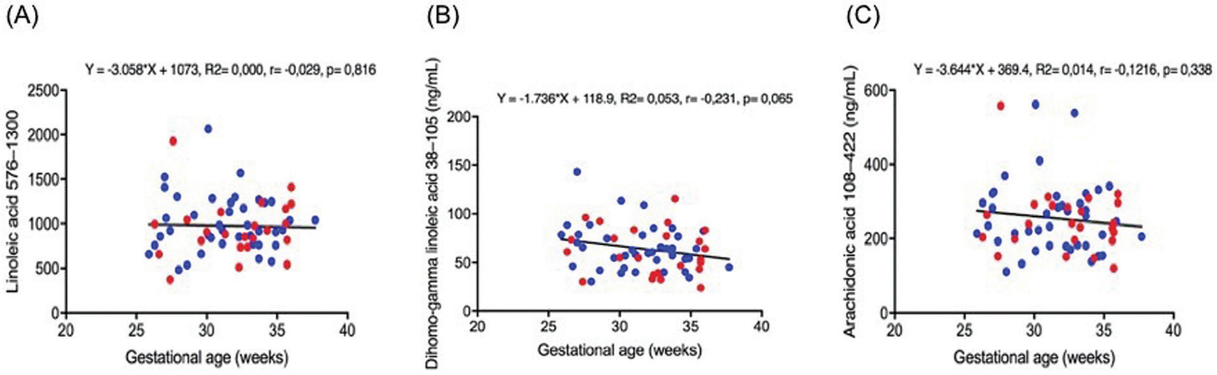

Supplementary Material Figure S3. Correlation between the omega-6 polyunsaturated fatty acid levels measured in pregnant women with appropriate for gestational age (blue dots), fetal growth restriction (red dots), and gestational age. Spearman test, $p < 0.05$

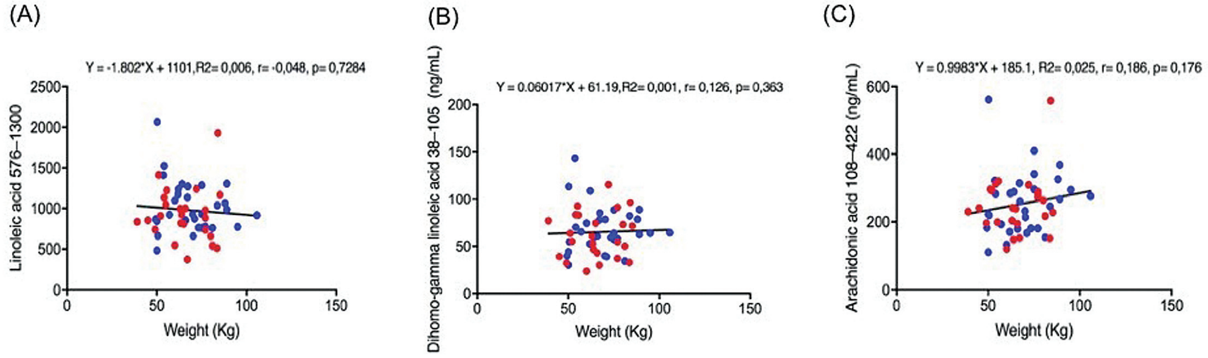

Supplementary Material Figure S4. Correlation between the omega-6 polyunsaturated fatty acid levels measured in pregnant women with appropriate for gestational age (blue dots), fetal growth restriction (red dots), and maternal weight. Spearman test, $p < 0.05$

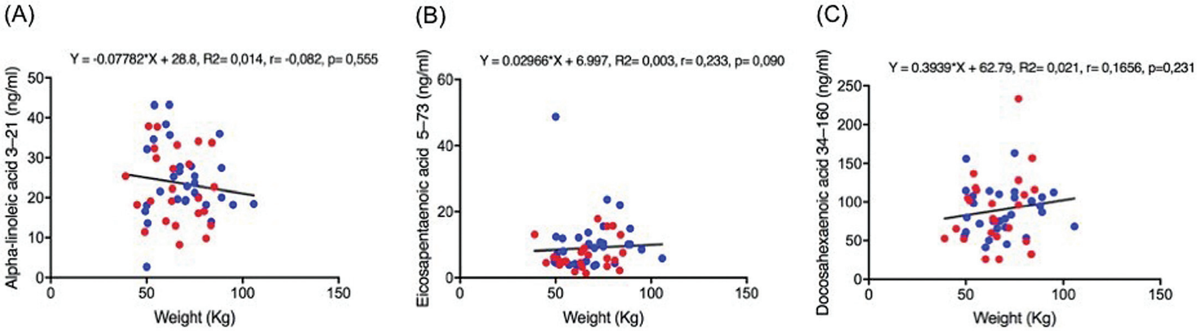

Supplementary Material Figure S5. Correlation between the omega-3 polyunsaturated fatty acid levels measured in pregnant women with appropriate for gestational age (blue dots), fetal growth restriction (red dots), and maternal weight. Spearman test, $p < 0.05$

 © 2023. Federação Brasileira de Ginecologia e Obstetrícia. All rights reserved.

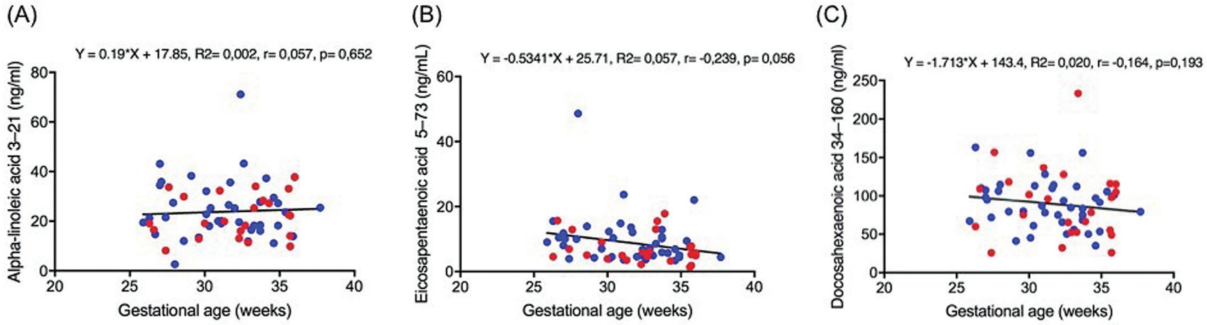

Supplementary Material Figure S6. Correlation between the omega-3 polyunsaturated fatty acid levels measured in pregnant women with appropriate for gestational age (blue dots), fetal growth restriction (red dots), and gestational age. Spearman test, $p < 0.05$

 © 2023. Federação Brasileira de Ginecologia e Obstetrícia. All rights reserved.
